# Association between secondhand smoke exposure and anxiety among adolescents: A nationwide cross-sectional study

**DOI:** 10.18332/tid/191750

**Published:** 2024-08-20

**Authors:** Jae Hyeok Lim, Dan Bi Kim, Jisu Ko, Min Jeong Joo, Eun-Cheol Park

**Affiliations:** 1 Graduate School of Public Health, Yonsei University, Seoul, Republic of Korea; 2 Institute of Health Services Research, Yonsei University, Seoul, Republic of Korea; 3 Department of Preventive Medicine and Public Health, College of Medicine, Yonsei University, Seoul, Republic of Korea

**Keywords:** secondhand smoke, generalized anxiety disorder, public place, exposure frequency, adolescent

## Abstract

**INTRODUCTION:**

Adolescents are especially susceptible to the harmful effects of secondhand smoke exposure. Mental health issues may be one of these negative consequences. This study aimed to explore the association between secondhand smoke exposure and anxiety among Korean adolescents.

**METHODS:**

This study used the nationwide representative cross-sectional data obtained from the 4-year (2020-2023) Korea Youth Risk Behavior Survey. In total, 214514 individuals aged 12–18 years were included in this study (109910 males and 104604 females). Secondhand smoke exposure was assessed based on responses to questions concerning the days they were exposed (at home and in public places), while anxiety levels were measured using the Generalized Anxiety Disorder 7 scale. Multivariable logistic regression analysis divided by sex was performed to explore the association (p=0.0173 for interaction by secondhand smoke exposure and sex), and adjusted odds ratios (ORs) with 95% confidence intervals (CI) were calculated.

**RESULTS:**

Among the study population, 8.8% of the male and 15.6% of the female participants had anxiety. After adjusting for covariates, adolescents with secondhand smoke exposure had a higher likelihood of reporting anxiety than those without the exposure (male, OR=1.23; 95% CI: 1.16–1.29; female, OR=1.27; 95% CI: 1.21–1.33). In additional stratified analyses, this association was more prominent among those who were never smokers, were exposed for more days, and had severe levels of anxiety.

**CONCLUSIONS:**

This study found that secondhand smoke exposure was associated with anxiety in adolescents; hence, proper political interventions to reduce secondhand smoke exposure may be required.

## INTRODUCTION

The detrimental impact of smoking on health is widely acknowledged^1^. Such effects directly affect smokers and indirectly impact others through exposure to secondhand smoke. Research indicates that secondhand smoke can have equally deleterious effects on human health as direct smoking^2^. The World Health Organization (WHO) emphasizes the risks of secondhand smoke and advocates for legislative measures to enforce smoke-free environments globally^3^. Despite these efforts, a substantial portion of the population continues to suffer from the negative consequences of secondhand smoke, with WHO statistics revealing that 1.3 million non-smokers die annually due to exposure^3^. Furthermore, a significant number of teenagers (12–16 years) worldwide are susceptible to secondhand smoke, particularly in public settings, with rates ranging from 68.2% in Korea to 57–72% depending on the economic level of each country^4^.

Adolescents are particularly vulnerable to the repercussions of exposure to secondhand smoke, given their pivotal stage of physical and mental development. The adverse mental effects of secondhand smoke on brain development are projected to be more severe in this age group than in adults^5^. Since children and adolescents tend to spend most of their time in residential and educational settings, they are at an increased risk of involuntary exposure to secondhand smoke^6^. Additionally, the absence of a safe threshold for exposure to secondhand smoke underscores the potential risks faced by adolescents, including a higher likelihood of subsequently engaging in smoking, which is a significant public health challenge^7,8^.

In light of these risks and conjectures, numerous studies have evaluated the correlation between secondhand smoke exposure and mental health issues among adolescents, primarily focusing on depression. However, the body of research in this area is relatively nascent, necessitating further scientific evidence^9^. Notably, insufficient attention has been directed toward examining the potential correlation between adolescent exposure to secondhand smoke and anxiety levels. Along with depression, anxiety is recognized as a prevalent mental disorder among adolescents^10^, with a prevalence of 11.2% in Korea and 7–17% globally, depending on area^11,12^. However, in contrast to depression, anxiety demonstrates differences, such as cognitive bias and attentional bias toward positive stimuli, as evidenced in research^13^. Consequently, the relationship between secondhand smoke exposure and associated factors may manifest differently in the context of anxiety.

A prior investigation indicated an association between secondhand smoke exposure and anxiety in South Korean adolescents using data from 2021^14^; however, additional research is warranted to ascertain the enduring effects, especially considering potential shifts due to evolving circumstances such as the coronavirus disease (COVID-19) pandemic^3,15^. Therefore, this study aimed to explore the association between secondhand smoke exposure and anxiety among Korean adolescents, utilizing nationwide cross-sectional data spanning 4 years, including the post-COVID-19 era.

## METHODS

### Data

This study used data from the Korea Youth Risk Behavior Survey (KYRBS), which was conducted by the Korea Disease Control and Prevention Agency. Each April, the national population of middle and high school students is targeted, with the KYRBS employing multistage stratified random cluster sampling to produce a nationally representative sample. To minimize sampling error, the sample is stratified into 117 strata based on 39 regional groupings and school levels. To match the population, 400 middle schools and 400 high schools were chosen for sample allocation each year using proportional sampling. The survey period consistently spans from August to October annually, with only a slight deviation of approximately one month during the COVID-19 pandemic. The response rates have remained high over the years, with 94.9% in 2020, 92.9% in 2021, 92.2% in 2022, and 92.9% in 2023. Participants respond anonymously to the online self-report questionnaire, and the involvement of others during the completion of the questionnaire is strictly controlled^16^.

### Participants

We utilized the 4-year period of KYRBS data (2020-2023) that initially included 214526 students, aged 12–18 years. Since KYRBS utilizes an online survey method that does not proceed to the next question if a question is not answered, there was no item ‘non-response’ in the original survey data; hence, 214514 individuals were eventually included in this study population, even after removing the missing values of relevant variables (109910 males and 104604 females). Ethics approval for using the KYRBS data was waived by the KCDC institutional review board under the Bioethics & Safety Act, as it is open to the public for academic use. All participants in the KYRBS, along with their parents or legal guardians, filled in informed consent forms.

### Variables

We obtained two questions to assess secondhand smoke exposure experienced by adolescents: 1) ‘How many times in the previous 7 days have you inhaled cigarette smoke from someone else in your household?’ and 2) ‘How many times in the previous 7 days have you inhaled cigarette smoke from someone else indoors (store, restaurant, shopping mall, venue, PC room, karaoke, etc.) rather than at home or school?’ with eight answer options ranging from 0 to 7 days a week. Those who replied at least 1 day to any of the two questions were defined as having experienced secondhand smoke exposure. For subgroups, the places exposed to secondhand smoke were classified as: 1) none, 2) home, 3) public place, and 4) both. Additionally, the days of secondhand smoke exposure for the two questions were summed and classified as: 1) none (0 days); 2) 1-3 days; 3) 4-5 days; 4) 6-7 days; and 5) ≥7 days.

Anxiety was measured using the Generalized Anxiety Disorder 7 (GAD-7) scale, which comprises seven items. The GAD-7 assesses the frequency of symptoms experienced by the examinees in the past 2 weeks and answers are rated on a 4-point Likert scale^17,18^. The reliability and validity of the GAD-7 has previously been reported among Korean participants^17,19^. The GAD-7 results were calculated using the sum of each answer score to a question: minimal (0–4), mild (5–9), moderate (10–14), and severe (15–21). Scores exceeding moderate (10–21) and below moderate (0–9) were assigned to the anxiety ‘yes’ and ‘no’ groups, respectively.

The following factors were included as covariates for potential confounders or risk factors: sex (male or female), grade (7th, 8th, 9th, 10th, 11th, or 12th), academic performance (high, middle, or low), area of residence (metropolitan, urban, or rural), residence type (living with or without family), economic status (high, middle, or low), subjective health condition (healthy, normal, or unhealthy), physical activity over 1 h and five times per week (yes or no), drinking experience (yes or no), smoking status (never, ex-smoker, or current smoker), sleep satisfaction (satisfied, regular, or unsatisfied), stress perception (high, middle, or low), depressive symptoms in the past 12 months (yes or no), and year (2020, 2021, 2022, or 2023).

### Statistical analysis

All analyses were stratified by sex owing to the significant interaction between secondhand smoke exposure and sex in the model (p=0.0173 for interaction), as well as differences in the impacts of smoking and anxiety^20,21^. For descriptive variables, frequency and percentage are presented with results of the chi-squared test to examine the general characteristics of the study population. Multivariable logistic regression analysis was used to explore the association between secondhand smoke exposure and anxiety among adolescents by applying regional strata, school clusters, and weighting values. Subgroup analyses, stratified by the place and frequency of secondhand smoke exposure and other independent variables, were conducted using multivariable logistic regression. The rising odds tendency across the ascending categories of the frequency of the secondhand smoke exposure was also examined in the model and the results are presented as the p-value for trend. To determine detailed associations, multinomial logistic regression was conducted with stratified GAD-7 scores. The associations between all variables are presented as odds ratios (ORs) and 95% confidence intervals (CIs). Statistical significance was determined at a two-sided p<0.05 and all statistical analyses were performed using SAS version 9.4M7 (SAS Institute, Cary, NC).

## RESULTS

Table 1 summarizes the general characteristics of the study population and the chi-squared test results for each independent and dependent variable. In total, 9659 (8.8%) of the 109910 male participants and 16365 (15.6%) of the 104604 female participants had anxiety. The chi-squared test revealed a significant association between secondhand smoke exposure and anxiety in males and females (p<0.0001).

**Table 1:** General characteristics of the study population in 2020–2023 KYRBS (N=214514)

*Characteristics*	*Male*							*Female*						
	*Total*		*Anxiety Yes*		*Anxiety No*		p*	*Total*		*Anxiety Yes*		*Anxiety No*		p*
	*n*	*%*	*n*	*%*	*n*	*%*		*n*	*%*	*n*	*%*	*n*	*%*	
	*109910*	*100*	*9659*	*8.8*	*100251*	*91.2*		*104604*	*100*	*16365*	*15.6*	*88239*	*84.4*	
**Secondhand smoke exposure**							<0.0001							<0.0001
No	56431	51.3	3994	7.1	52437	92.9		41508	39.7	4860	11.7	36648	88.3	
Yes	53479	48.7	5665	10.6	47814	89.4		63096	60.3	11505	18.2	51591	81.8	
**Grade**							<0.0001							<0.0001
7th	19949	18.2	1432	7.2	18517	92.8		18953	18.1	2681	14.1	16272	85.9	
8th	19591	17.8	1571	8.0	18020	92.0		18897	18.1	3054	16.2	15843	83.8	
9th	19441	17.7	1782	9.2	17659	90.8		18554	17.7	3046	16.4	15508	83.6	
10th	17819	16.2	1569	8.8	16250	91.2		17085	16.3	2559	15.0	14526	85.0	
11th	17383	15.8	1634	9.4	15749	90.6		16295	15.6	2524	15.5	13771	84.5	
12th	15727	14.3	1671	10.6	14056	89.4		14820	14.2	2501	16.9	12319	83.1	
**Academic performance**							<0.0001							<0.0001
High	42001	38.2	3467	8.3	38534	91.7		38762	37.1	5484	14.1	33278	85.9	
Middle	32207	29.3	2399	7.4	29808	92.6		32304	30.9	4353	13.5	27951	86.5	
Low	35702	32.5	3793	10.6	31909	89.4		33538	32.1	6528	19.5	27010	80.5	
**Region**							<0.0001							0.0002
Metropolitan	47490	43.2	4008	8.4	43482	91.6		44978	43.0	6799	15.1	38179	84.9	
Urban	53773	48.9	4946	9.2	48827	90.8		51768	49.5	8303	16.0	43465	84.0	
Rural	8647	7.9	705	8.2	7942	91.8		7858	7.5	1263	16.1	6595	83.9	
**Residence type**							<0.0001							<0.0001
Living with family	104194	94.8	9007	8.6	95187	91.4		100108	95.7	15505	15.5	84603	84.5	
Living without family	5716	5.2	652	11.4	5064	88.6		4496	4.3	860	19.1	3636	80.9	
**Economic status**							<0.0001							<0.0001
High	47067	42.8	3770	8.0	43297	92.0		40136	38.4	5568	13.9	34568	86.1	
Middle	49854	45.4	3997	8.0	45857	92.0		51742	49.5	7527	14.5	44215	85.5	
Low	12989	11.8	1892	14.6	11097	85.4		12726	12.2	3270	25.7	9456	74.3	
**Health condition**							<0.0001							<0.0001
Healthy	77654	70.7	4572	5.9	73082	94.1		63218	60.4	6333	10.0	56885	90.0	
Normal	23464	21.3	2855	12.2	20609	87.8		30284	29.0	5817	19.2	24467	80.8	
Unhealthy	8792	8.0	2232	25.4	6560	74.6		11102	10.6	4215	38.0	6887	62.0	
**Physical activity**							0.0673							<0.0001
No	84732	77.1	7519	8.9	77213	91.1		95280	91.1	14689	15.4	80591	84.6	
Yes	25178	22.9	2140	8.5	23038	91.5		9324	8.9	1676	18.0	7648	82.0	
**Drinking experience**							<0.0001							<0.0001
No	68561	62.4	5250	7.7	63311	92.3		74768	71.5	10144	13.6	64624	86.4	
Yes	41349	37.6	4409	10.7	36940	89.3		29836	28.5	6221	20.9	23615	79.1	
**Smoking status**							<0.0001							<0.0001
Never	94570	86.0	7760	8.2	86810	91.8		97236	93.0	14307	14.7	82929	85.3	
Ex-smoker	8422	7.7	956	11.4	7466	88.6		4082	3.9	1036	25.4	3046	74.6	
Current smoker	6918	6.3	943	13.6	5975	86.4		3286	3.1	1022	31.1	2264	68.9	
**Sleep satisfaction**							<0.0001							<0.0001
Satisfied	33622	30.6	1485	4.4	32137	95.6		21709	20.8	1643	7.6	20066	92.4	
Regular	37651	34.3	2493	6.6	35158	93.4		33360	31.9	3803	11.4	29557	88.6	
Unsatisfied	38637	35.2	5681	14.7	32956	85.3		49535	47.4	10919	22.0	38616	78.0	
**Stress perception**							<0.0001							<0.0001
High	34614	31.5	7583	21.9	27031	78.1		46386	44.3	14156	30.5	32230	69.5	
Middle	49224	44.8	1789	3.6	47435	96.4		43886	42.0	2028	4.6	41858	95.4	
Low	26072	23.7	287	1.1	25785	98.9		14332	13.7	181	1.3	14151	98.7	
**Depressive symptom**							<0.0001							<0.0001
No	85853	78.1	3804	4.4	82049	95.6		71341	68.2	4963	7.0	66378	93.0	
Yes	24057	21.9	5855	24.3	18202	75.7		33263	31.8	11402	34.3	21861	65.7	
**Year**							<0.0001							<0.0001
2020	28353	25.8	2191	7.7	26162	92.3		26595	25.4	3908	14.7	22687	85.3	
2021	28401	25.8	2561	9.0	25840	91.0		26447	25.3	4144	15.7	22303	84.3	
2022	26393	24.0	2529	9.6	23864	90.4		25452	24.3	4058	15.9	21394	84.1	
2023	26763	24.3	2378	8.9	24385	91.1		26110	25.0	4255	16.3	21855	83.7	

* The p-values were estimated using chi-squared test.

Table 2 presents the results of the multivariable logistic regression analysis of secondhand smoke exposure and anxiety. Compared with adolescents who had no secondhand smoke exposure, adolescents exposed to secondhand smoke had a significantly higher likelihood of anxiety (male, OR=1.23; 95% CI: 1.16–1.29; female, OR=1.27; 95% CI: 1.21–1.33) after adjusting for all covariates.

**Table 2:** Results of factors associated between secondhand smoke exposure and anxiety in 2020–2023 KYRBS (N=214514)

*Variables*	*Male*		*Female*	
	*OR*	*95% CI*	*OR*	*95% CI*
**Secondhand smoke exposure**				
No ®	1		1	
Yes	1.23	1.16–1.29	1.27	1.21–1.33
**Grade**				
7th ®	1		1	
8th	1.06	0.97–1.16	1.03	0.96–1.11
9th	1.14	1.04–1.25	0.97	0.90–1.05
10th	1.02	0.92–1.13	0.77	0.71–0.83
11th	1.08	0.98–1.19	0.75	0.69–0.81
12th	1.20	1.09–1.32	0.88	0.81–0.95
**Academic performance**				
High ®	1		1	
Middle	0.88	0.82–0.94	0.89	0.84–0.94
Low	0.98	0.92–1.05	1.03	0.98–1.09
**Region**				
Metropolitan ®	1		1	
Urban	1.09	1.03–1.15	1.04	0.99–1.09
Rural	0.95	0.86–1.05	1.01	0.93–1.10
**Residence type**				
Living with family ®	1		1	
Living without family	1.18	1.05–1.32	1.15	1.03–1.28
**Economic status**				
High ®	1		1	
Middle	0.92	0.87–0.97	0.98	0.94–1.03
Low	1.14	1.06–1.22	1.35	1.27–1.44
**Health condition**				
Healthy ®	1		1	
Normal	1.54	1.45–1.63	1.39	1.33–1.46
Unhealthy	2.62	2.44–2.82	2.59	2.44–2.74
Physical activity				
No ®	1		1	
Yes	1.02	0.96–1.08	1.12	1.04–1.20
**Drinking experience**				
No ®	1		1	
Yes	0.98	0.92–1.04	1.06	1.01–1.11
**Smoking status**				
Never ®	1		1	
Ex-smoker	1.06	0.97–1.16	1.19	1.08–1.31
Current smoker	0.97	0.88–1.06	1.34	1.21–1.48
**Sleep satisfaction**				
Satisfied ®	1		1	
Regular	1.07	0.99–1.16	1.10	1.02–1.19
Unsatisfied	1.61	1.50–1.73	1.53	1.43–1.64
**Stress perception**				
High ®	1		1	
Middle	0.22	0.21–0.24	0.19	0.18–0.20
Low	0.09	0.08–0.10	0.07	0.06–0.09
**Depressive symptom**				
No ®	1		1	
Yes	3.54	3.34–3.74	3.57	3.42–3.73
**Year**				
2020 ®	1		1	
2021	1.00	0.92–1.08	0.93	0.87–0.99
2022	0.92	0.86–1.00	0.88	0.82–0.94
2023	1.01	0.93–1.09	1.05	0.98–1.12

OR and 95% CI were estimated using multivariable logistic regression with adjustment for all covariates.

® Reference categories.

Table 3 presents the results of the subgroup analysis stratified by independent variables. Adolescents who did not smoke but who were exposed to secondhand smoke had significantly higher odds of anxiety than those without secondhand smoke exposure in both males and females. Regarding mental health variables, more prominent associations were observed in the healthy categories of sleep satisfaction, stress perception, and depressive symptoms.

**Table 3:** Results of subgroup analysis stratified by independent variables

*Variables*	*Male*				*Female*			
	*Anxiety No*	*Anxiety Yes*		*p for interaction*	*Anxiety No*	*Anxiety Yes*		*p for interaction*
	*OR*	*OR*	*95% CI*		*OR*	*OR*	*95% CI*	
**Smoking status**								
Never	1	1.24	1.17–1.31	Ref.	1	1.28	1.22–1.35	Ref.
Ex-smoker	1	1.28	1.07–1.52	0.2848	1	1.22	1.00–1.49	0.4542
Current smoker	1	1.03	0.85–1.24	0.0842	1	0.92	0.74–1.14	0.0306
**Sleep satisfaction**								
Satisfied	1	1.25	1.11–1.41	Ref.	1	1.43	1.26–1.63	Ref.
Regular	1	1.28	1.16–1.42	0.5857	1	1.17	1.07–1.28	0.0079
Unsatisfied	1	1.19	1.11–1.28	0.3191	1	1.27	1.20–1.35	0.9273
**Stress perception**								
High	1	1.20	1.12–1.27	Ref.	1	1.24	1.18–1.31	Ref.
Middle	1	1.32	1.19–1.47	0.5367	1	1.33	1.19–1.47	0.5028
Low	1	1.20	0.92–1.56	0.5870	1	1.42	1.01–1.99	0.1178
**Depressive symptom**								
No	1	1.28	1.18–1.37	Ref.	1	1.32	1.23–1.42	Ref.
Yes	1	1.18	1.10–1.27	0.0917	1	1.22	1.15–1.30	0.0658

OR and 95% CI were estimated using multivariable logistic regression with adjustment for all covariates, except for each stratified variable.P-values for interaction were estimated between secondhand smoke exposure and each stratified variable in the model.

® Reference categories.

**Figure 1:**
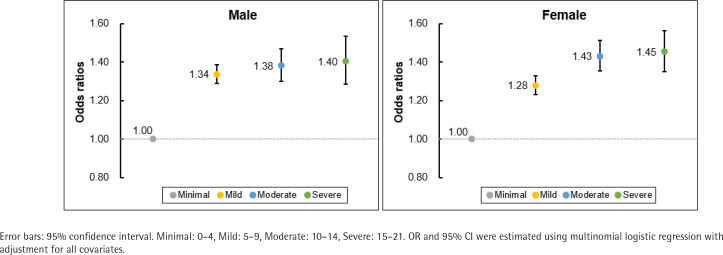
Results of analysis stratified by Generalized Anxiety Disorder-7 score in 2020–2023 KYRBS (N=214514)

Figure 1 displays the results stratified by GAD-7 score categories using multinomial logistic regression. Adolescents with secondhand smoke exposure exhibited gradually higher odds in the order of mild (male, OR=1.34; 95% CI: 1.29–1.39; female, OR=1.28; 95% CI: 1.23–1.33), moderate (male, OR=1.38; 95% CI: 1.30–1.47; female, OR=1.43; 95% CI: 1.35–1.51), and severe (male, OR=1.40; 95% CI: 1.29–1.53; female, OR=1.45; 95% CI: 1.35–1.57) scores than the minimal score without secondhand smoke exposure.

**Figure 2:**
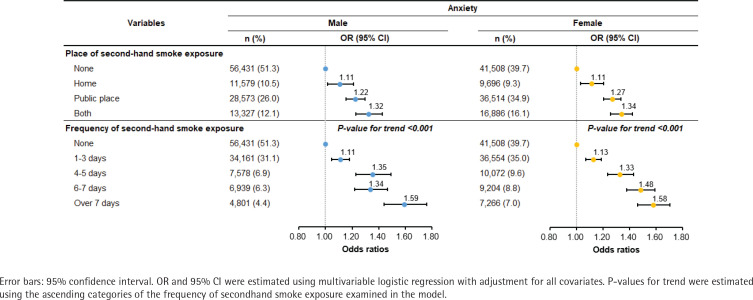
Results of subgroup analysis stratified by secondhand smoke exposure in 2020–2023 KYRBS (N=214514)

Figure 2 displays the results stratified by the place and frequency of secondhand smoke exposure. In the stratification analysis by place, adolescents exposed to secondhand smoke at their home (male, OR=1.11; 95% CI: 1.01–1.21; female, OR=1.11; 95% CI: 1.03–1.20) and in public places (male, OR=1.22; 95% CI: 1.15–1.30; female, OR=1.27; 95% CI: 1.21–1.34) had a higher likelihood of anxiety. Secondhand smoke exposure in both places exhibited a higher likelihood of anxiety (male, OR=1.32; 95% CI: 1.23–1.43; female, OR=1.34; 95% CI: 1.26–1.42). In stratification analysis by frequency, adolescents who were exposed to secondhand smoke more days in a week had a higher likelihood of anxiety with a linear trend in males (1-3 days, OR=1.11; 95% CI: 1.05–1.18; 4-5 days, OR=1.35; 95% CI: 1.23–1.49; 6-7 days, OR=1.34; 95% CI: 1.22–1.47; over 7 days, OR=1.59; 95% CI: 1.44–1.76; p<0.001 for trend) and in females (1-3 days, OR=1.13; 95% CI: 1.07–1.19; 4-5 days, OR=1.33; 95% CI: 1.23–1.43; 6–7 days, OR= 1.48; 95% CI: 1.38–1.59; over 7 days, OR=1.58; 95% CI: 1.46–1.71; p<0.001 for trend).

## DISCUSSION

This study examined the association between secondhand smoke exposure and anxiety in representative South Korean adolescents aged 12–18 years. After adjusting for potential confounders, we found that secondhand smoke exposure in adolescents was significantly associated with anxiety. The GAD-7 score tended to increase linearly compared with the minimal level. More days of secondhand smoke exposure were also linearly associated with a higher likelihood of anxiety.

As indicated by the outcomes of this investigation, the majority of prior research that indicated an association between secondhand smoke exposure and anxiety focused on non-smoking adolescents^9,22-24^, with only one study encompassing both smokers and non-smokers^25^. The comparison with non-smoking adolescents, including adolescent smokers in the study population, is important since it takes into account surroundings based on smoking status^25^. Despite a slight variance in the participants’ ages, the effect size of the association observed in previous studies was similar to that of this study. Regarding this association, possible biological mechanisms elucidating the impact of smoking on anxiety include an array of processes, including the dopaminergic system, gamma-aminobutyric acid, hypothalamic-pituitary-adrenal axis, serotonin, and monoamine oxidase^26-28^. Given that adolescence is a critical period in mental development, exposure to secondhand smoke may have an impact on the occurrence of mental health issues in adolescents.

Nonetheless, a conclusive mechanism linking secondhand smoke with mental health, including anxiety, is yet to be clearly identified. While a notable age gap exists between the participants in this study – adults and individuals aged 5-12 years – other research exploring the association between exposure to secondhand smoke and anxiety yielded contradicting results, hinting at alternative potential mechanisms^27,29^. Socioeconomic factors might have affected the outcomes of this association since they are unmeasured confounding variables^30^. Indeed, various factors, such as low income or poor environmental settings conducive to mental illness, may influence the act of smoking^31,32^. Analogous to active smoking, environments permeated with secondhand smoke may also be generally correlated with lower level of parental education and financially challenging circumstances^33^. Consequently, the ambient environment characterized by secondhand smoke might have impacted mental health, or both secondhand smoke and the environment as influencing factors could have contributed in an additive manner. Nevertheless, in both anxiety levels and the effects of secondhand smoke, the dose-response association identified in this study suggests that secondhand smoke may elucidate at least a portion of adolescent anxiety.

In a subgroup analysis where smoking status was stratified, smokers did not exhibit a significant association between secondhand smoke exposure and anxiety. Smokers may display an elevated susceptibility to anxiety, irrespective of exposure to secondhand smoke, due to the direct inhalation of elements that influence anxiety levels^34^. Alternatively, in line with self-medication theory, engaging in smoking habits while experiencing psychological instability can lead to temporary alleviation of anxiety symptoms^35^. This suggests that smoking could be a strategy adopted by individuals to manage their anxiety levels in the short-term. Conversely, former smokers and non-smokers showed a significant correlation. This finding can be elucidated by studies indicating that non-smokers exposed to secondhand smoke may experience heightened sensitivity to stress and pain^22^. Unlike depression, which is characterized by a tendency to recall negative memories and a diminished response to positive stimuli, anxiety differs by not avoiding or suppressing positive stimuli^13^. Supporting this notion, the current study confirms that the likelihood of anxiety stemming from secondhand smoke exposure can be substantial even among adolescents who report satisfactory sleep, minimal stress, and an absence of depressive symptoms.

These findings hold implications for addressing the mental health challenges faced by adolescents via policy measures, particularly regarding the diverse activities engaged in by adolescents, such as exposure to secondhand smoke in various public settings, which can influence the prevalence of anxiety^22^. Further research may elucidate the underlying mechanisms between secondhand smoke exposure and anxiety from a longitudinal viewpoint while considering any potential confounding variables that may impact the observed associations. This would contribute to a more comprehensive understanding of the complex interplay between environmental factors like secondhand smoke and the development of anxiety among adolescents, thereby informing more targeted and effective interventions in the realm of mental health promotion.

### Strengths and limitations

The strengths of this study are that we performed anxiety screening of adolescents using the validated GAD-7 tool and that the findings can be generalized to all South Korean adolescents because nationally representative data were used. However, this study had some limitations and thus requires careful interpretation. First, this study used a cross-sectional design; therefore, it was difficult to clarify the temporal relationships between the variables. Second, the self-reporting approach of KYRBS (including recalling memories of secondhand smoke experiences in a week) may have introduced bias. Additionally, due to the absence of biological measurements, there may be a difference in the intensity of exposure even when the same exposure duration is used to respond to secondhand smoking. Third, this study was used by pooling data from four years (2020–2023) to include information after the COVID-19 pandemic, which could impact the mental health of adolescents. Thereby, it is possible that there might be instances of heterogeneity in the variables utilized within each specific cross-sectional unit, potentially leading to inadequacies despite the adjustment of year clusters made in the model to account for such issues. Fourth, differences in the prevalence of secondhand smoke exposure and anxiety among adolescents vary across different regions globally. If there are specific socio-cultural factors alongside biological processes influencing this relationship, various countries may exhibit divergent outcomes. Lastly, confounders that affect anxiety may not have been fully adjusted for because they were not included in the survey. For example, information on a mental illness diagnosis before the survey or family history may need to be adjusted.

## CONCLUSIONS

This study found that secondhand smoke exposure was significantly associated with anxiety in adolescents, as measured using the GAD-7 scale. Additionally, this association increased as exposure to secondhand smoke and anxiety levels rose. This suggests that proper political interventions to reduce secondhand smoke exposure may be required in areas where adolescents are active.

## Data Availability

The data supporting this research can be found in the Korea Centers for Disease Control and Prevention Agency, KYRBS website (https://www.kdca.go.kr/yhs/home.jsp).
